# Les carcinomes de la thyroïde: profils épidémiologique, clinique et thérapeutique, à propos de 102 cas

**DOI:** 10.11604/pamj.2015.21.59.5688

**Published:** 2015-05-26

**Authors:** Mohamed Mliha Touati, Abdelfettah Aljalil, Youssef Darouassi, Mehdi Chihani, Mohammed Lahkim, Jawad Al Fassi Fihri, Brahim Bouaity, Haddou Ammar

**Affiliations:** 1 Service d'Oto-rhino-laryngologie et Chirurgie Cervico-faciale, Hôpital Militaire Avicenne, Marrakech, Maroc; 2 Service de Chirurgie Viscérale, Hôpital Militaire Avicenne, Marrakech, Maroc

**Keywords:** Carcinome thyroïdien, chirurgie, anatomo-pathologie, thyroid carcinoma, surgery, anatomy-pathology

## Abstract

Les carcinomes thyroïdiens sont des tumeurs malignes assez rares, représentant 1% des cancers. Ils sont généralement de bon pronostic, et présentent des aspects cliniques et évolutifs variés selon leur origine histologique. Notre travail est une étude rétrospective portant sur 102 cas de carcinomes de la thyroïde, colligés sur un nombre total de 811 gestes chirurgicaux thyroïdiens, au service d'oto-rhino-laryngologie et de chirurgie cervico-faciale de l'hôpital militaire Avicenne de Marrakech, sur une période de 8 ans, allant de janvier 2006 à décembre 2013. Les carcinomes thyroïdiens atteignent le sujet jeune avant l’âge de 50 ans, en particulier le sexe féminin. La tendance dans les pays en voie de développement, comme dans le monde entier est en croissance continue, ceci peut être expliqué par l'amélioration des outils d'imagerie et des moyens diagnostiques cytologiques et anatomo-pathologiques.

## Introduction

Les carcinomes thyroïdiens sont des tumeurs malignes assez rares, représentant 1% des cancers. Ils sont généralement de bon pronostic, et présentent des aspects cliniques et évolutifs variés selon leur origine histologique [1]. Depuis les années 1970, l'incidence du cancer thyroïdien augmente dans la plupart des pays, dont le Maroc où elle est estimée à 0,6/100 000 [2]. Cette augmentation peut être expliquée par la performance et le faible coût des moyens diagnostiques (échographie, cytoponction), par la sensibilisation du milieu médical et de la population générale, le suivi des personnes à haut risque et la modification des critères histologiques, comme en témoigne la proportion croissante des formes papillaires avec microcancers [2]. Le but de notre étude est de préciser le profil épidémiologique, l'expression clinique, le diagnostic histologique, les moyens thérapeutiques, ainsi que l’évolution et le pronostic des carcinomes de la thyroïde.

## Méthodes

Notre travail est une étude rétrospective portant sur 102 cas de carcinomes de la thyroïde, colligés sur un nombre total de 811 gestes chirurgicaux thyroïdiens, au service d'oto-rhino-laryngologie et de chirurgie cervico-faciale de l'hôpital militaire Avicenne de Marrakech, sur une période de 8 ans, allant de janvier 2006 à décembre 2013. Les carcinomes inclus dans notre étude, ont été sélectionné à partir des dossiers contenant une preuve histologique, et sur la base de la classification histologique des tumeurs thyroïdiennes proposée par l'organisation mondiale de la santé (OMS) en 2004 [3]. Notre travail a exclu les cancers thyroïdiens d'origine non épithéliale, les cancers régionaux envahissant la thyroïde, les métastases secondaires, et les tumeurs bénignes. Les renseignements cliniques, paracliniques et évolutifs ont été recueillis à partir des dossiers, et du suivi des malades en consultation.

## Résultats

**Figure 1:**
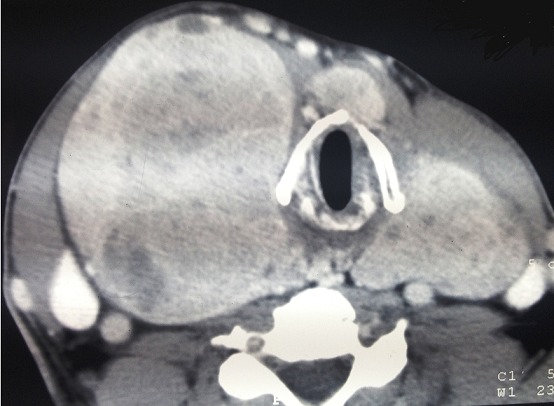
TDM cervicale en coupes axiales montrant une tumeur thyroïdienne avec prise de contraste hétérogène

**Figure 2:**
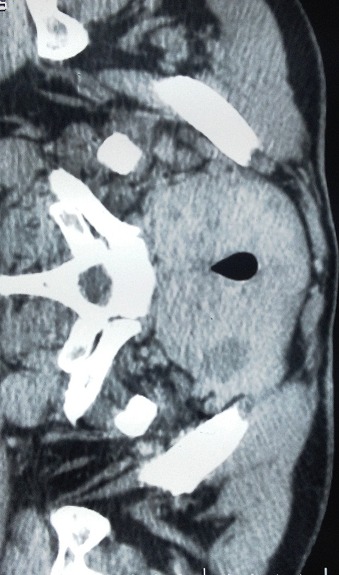
TDM cervicale en coupe axiale montrant tumeur thyroïdienne, avec compression trachéale

Notre série comprend 76% de femmes et 24% d'hommes. La moyenne d’âge chez les hommes est de 56,33 ans, et de 42,42 ans pour les femmes. La moyenne d’âge dans les deux sexes est de 45,76 ans, avec des extrêmes allant de 27 à 69 ans (Tableau 1). Aucun de nos patient n'a était victime d'une irradiation accidentelle ou iatrogène, et aucun cas de carcinome thyroïdien familial n'a été rapporté. Le motif de consultation le plus fréquent est une masse cervicale antérieure asymptomatique, retrouvé dans 76% des cas, la durée d’évolution était supérieure à 2 ans dans 60% des cas. L'examen clinique retrouve un nodule thyroïdien solitaire (70%), un goitre multinodulaire (18%), et un goitre homogène (12%). L'examen clinique a également retrouvé des signes de dysthyroïdie chez 14% des patients, des adénopathies cervicales supérieures à 1 cm de diamètre, dans 6% des cas. L’échographie cervicale a révélée un goitre multi-hétéro-nodulaire chez 64% des malades, un nodule unique chez 36%, 47% des nodules thyroïdiens révélés sont hypoéchogènes. La taille échographique des nodules était comprise entre 14 et 75 mm de grand axe, avec une moyenne de 30,4mm. Les contours nodulaires n'ont été explorés que dans 60% des cas, ils étaient irréguliers dans 40% et flous dans 20% des cas. L’échographie a permis de retrouver des microcalcifications chez 27% des patients, et une hyper-vascularisation nodulaire dans 34% des cas. Le scanner cervical a été réalisé chez 35 patients (34,31%), en cas de goitre volumineux avec des signes de compression (Figure 1 et Figure 2). La cytoponction a été réalisée chez 34% des patients. Les résultats correspondaient à des lésions carcinomateuses papillaires suspectes dans 11% des cas. À la scintigraphie thyroïdienne (demandée systématiquement chez les patients présentant une dysthyroïdie), 16% de nos malades avaient des nodules froids, et 6% avaient des nodules thyroïdiens chauds. L'examen extemporané a été fait chez 24% de nos malades, et a permis de retrouver, des microcarcinomes papillaires dans 14% des cas, des lésions suspects d'architecture vésiculo-papillaire dans 8%, et un adénome microvésiculaire (faux-négatif) dans 2% des cas. L'examen histologique définitif, a révélé la présence de carcinomes papillaires dans 90% des cas (dont 38% représente la variante micropapillaire), de carcinomes vésiculaires dans 4%, de carcinomes peu-différenciés dans 3%, de carcinomes médullaires dans 2% des cas et de cancers anaplasiques dans 1%.

**Tableau 1:** Moyenne d’âge globale (M.A), chez les femmes et les hommes, en fonction des études

Etude	Moyenne d’âge	Femme	Homme
Gomez-Segovia	-	53	57
Fauconnier	46	46	53
Sassolas	-	50	49
Notre série	45,76	42,42	56,33

La thyroïdectomie totale a été réalisée chez 99% de nos patients, elle peut se répartir dans notre étude en 3 modalités: 1) thyroïdectomie totale d'emblé, a été effectuée dans 63% des cas; 2) totalisation après examen extemporané en faveur de la malignité, a été effectuée dans 22% des cas; et enfin, 3) la totalisation chirurgicale ultérieure après examen anatomo-pathologique définitif en faveur de la malignité, a été faite dans 14% des cas. Une chirurgie de réduction a été effectuée dans un cas (soit 1%) correspondant à un cancer anaplasique. Le curage ganglionnaire des chaines cervicales n'a été pratiqué que chez les malades présentant des adénopathies cliniques ou échographiques. Il intéressait les chaines ganglionnaires récurrentiels et jugulo-carotidiennes homolatérales. Les suites post-opératoires étaient sans particularité, en dehors d'une hypocalcémie transitoire retrouvée chez 16% des patients, l’évolution était rapidement favorable. L'irathérapie à l'iode 131, a été prescrite dans 54% des cas, à la dose de 100 mCi, pour les malades ayant des carcinomes de souches folliculaires. Quant à ceux porteurs de carcinomes anaplasiques ou médullaires, ils ont été adressés pour une radio-chimiothérapie adjuvante. Une enquête familiale a été effectuée chez les patients présentant le carcinome médullaire, et elle s'est avérée négative. L’évolution à long terme après le traitement, a été marquée par l'absence de toute récidive chez les patients qui avaient des cancers différenciés. Les malades porteurs de cancer médullaire n'ont pas présenté de récidive après une année de contrôle. Cependant l’évolution était fatale pour le malade porteur de cancer anaplasique, 6 mois après la radio-chimiothérapie.

## Discussion

Notre étude note une prédominance féminine avec un sex-ratio femme/homme de 3,17. Ce résultat est proche de ceux des séries africaines [3] et mondiales [4, 5]. La moyenne d’âge rejoint également celle des autres études [2–4]: elle se situe dans la 4^ème^ décennie, La moyenne d’âge (M.A) en fonction du sexe dans les différentes études est représentée dans le Tableau 1. Dans notre période d’étude, la prévalence des carcinomes thyroïdiens représentait 12,57%, Rego-Iraeta et al [6] rapportent un pourcentage similaire, alors que les donnés africaines [2, 4], rapportent une prévalence de 6,33% et 7,68% respectivement. La taille moyenne tumorale de 16 mm, reflète la sensibilité diagnostic des outils échographique et anatomopathologique de cette série, chose rapportée également par Xiang [7]. Les données de la littérature présente une nette prédominance des carcinomes thyroïdiens différenciés de souche folliculaire (papillaires et vésiculaires), leur prévalence dans notre étude est de 94%, elle est similaire à celle des études mondiales de Rego-Iraeta [6] (89,7%), Mehry [8] (94%), et de Xiang [7] (96,6%). Par contre, le profil épidémiologique de chacun des deux types histologiques diffère d'une série à l'autre: les cancers vésiculaires étaient moins fréquents dans notre étude (4%) par rapport à la plupart des fréquences rapportées dans la littérature. Leur prévalence était de 45% dans deux séries africaines [2, 4], par contre elle était dans les séries mondiales de Sassolas [9], Fauconnier [10], Rego-Iraeta [6], et de Brownlie [11], de l'ordre de 5.9%, 11%, 13.7%, et de 19% respectivement. En effet, la carence en iode, sévissant en Afrique, est incriminée dans la survenue de ce type de cancer [4]; Les carcinomes papillaires, quant à eux, avaient une fréquence de 90% dans notre étude, la plaçant au cotés de séries mondiales récentes de Xiang [7] (92,8%) et de Sassolas [9] (86.5%), qui ont toutes comme point commun une part importante des microcarcinomes (38% dans notre étude, 35,7% chez Xiang et 36% chez Sassolas), révélés par l'examen anatomopathologique à l'occasion de thyroïdectomies pour des pathologies bénignes. Par ailleurs, la prévalence des carcinomes papillaires dans la série africaine de Rakotoarisoa [3] sont de 50%. Pratiquement tous nos malades ont bénéfices d'une thyroïdectomie totale en un ou deux temps. Les recommandations de «l'American Thyroid Association» (ATA) [12] sont en faveur de cette pratique chirurgicale, non pas seulement pour les tumeurs supérieures à 1 cm, mais aussi pour celles inférieures à 1 cm également, compte-tenu de la présence de certains facteurs associés, pouvant favoriser leurs récidives; parmi eux: l’âge > 45 ans, les microcarcinomes plurifocaux (16% dans notre étude).

## Conclusion

Les carcinomes thyroïdiens atteignent le sujet jeune avant l’âge de 50 ans, en particulier le sexe féminin. La tendance dans les pays en voie de développement, comme dans le monde entier est en croissance continue, ceci peut être expliqué par l'amélioration des outils d'imagerie et des moyens diagnostiques cytologiques et anatomo-pathologiques. Grâce à ces moyens, une grande partie de nos malades, porteurs de microcarcinomes, ont bénéficiés d'une thyroïdectomie totale, leur épargnant une évolution vers un stade avancé de la maladie.
